# Anesthesia can alter the levels of corticosterone and the phosphorylation of signaling molecules

**DOI:** 10.1186/s13104-021-05763-w

**Published:** 2021-09-19

**Authors:** Gabriela Cruz Pereira, Marcos Mônico Neto, Hanna Karen Moreira Antunes, Kil Sun Lee, Marcio Henrique Mello da Luz

**Affiliations:** 1 Department of Biochemistry, Federal University of São Paulo, Rua Pedro de Toledo 669, 8° andar, Vila Clementino, São Paulo, SP Brazil; 2 Department of Psychobiology, Federal University of São Paulo, São Paulo, SP Brazil

**Keywords:** Ketamine, Xylazine, Sleep deprivation, Corticosterone, Signaling molecules

## Abstract

**Objective:**

Neuroscience research using laboratory animals has increased over the years for a number of reasons. Some of these studies require the use of anesthetics for surgical procedures. However, the use of anesthetics promotes several physiological changes that may interfere with experimental results. Although the anesthetics and methods of delivery used to vary, one of the most common is ketamine associated with another compound such as xylazine. We aimed to evaluate the effect of ketamine and xylazine (KX) on corticosterone levels and on the degree of phosphorylation of p44/42 (ERK1/2), Src kinases and calcium/calmodulin-dependent kinase II (CAMKII). We also compared the effects of KX on sleep deprivation, which is known to affect the hormonal profile including corticosterone.

**Results:**

We found that the use of KX can increase corticosterone levels and alter the degree of phosphorylation of signaling proteins.

**Supplementary Information:**

The online version contains supplementary material available at 10.1186/s13104-021-05763-w.

## Introduction

Rodents have been widely used in neurobiological studies for better understanding brain structure, physiology and function [1], including developmental processes, cognitive function, pathogenesis and the progression of psychiatric and neurological disorders [2, 3]. Along with that, surgical techniques are used for obtainment of cerebrospinal fluid samples, implantation of cannula and electrodes [4]. These interventions require anesthesia and analgesia during procedures [5, 6], that can directly impact metabolic pathways as well as levels of nucleotides, nucleosides, lipids, amino acids, and blood and metabolic serum biomarkers like corticosterone [7–9].

Most common anesthetics used in research are injectable pentobarbital or ketamine combined with other agents such as xylazine (KX) [6]. Ketamine acts as an antagonist for NMDA receptor (NMDAR) blocking excitatory synaptic activity and blocking Na^+^ channels in high-dose [10, 11]. Xylazine is an adrenergic α2 receptors agonist that decreases norepinephrine and dopamine release resulting in muscle relaxation and sedation [12].

Some published data have suggested that combination of KX may alter hormone levels [13, 14]. Saha et al. showed a reduction in corticosterone levels by 55% after 30 min of anesthesia and 34% after 180 min in fasted rats for 18-h. In fed rats, a reduction of 34% after 120 min, and 51% after 180 min of anesthesia [13]. On the other hand, Chen et al. reported an increase in fasted rats after 60 and 120 min [14]; Arnold and Langhans in counterpart showed no difference in corticosterone levels after anesthesia [8]. There is, therefore, a clear divergence regarding the effects of KX on corticosterone levels.

Moreover, anesthetics in general are modulators of neuronal activity and, depending on the dosage and exposure time, they may affect several functions of brain such as neurogenesis, synaptic transmission and cell survival [15, 16]. These effects of anesthetic can be caused due to hippocampal alterations and malfunction [17]. Some signaling proteins perform essential roles in hippocampus: Src family kinases contributes to long-term potentiation and myelination [18], p44/42 MAP kinase (ERK1/2) participates in synaptic plasticity and spatial learning [19] and Ca^2+^/calmodulin-dependent protein kinase II (CaMKII) acts on synaptic plasticity and memory [20, 21].

Similar to anesthesia, sleep loss can also disrupt the homeostasis of several physiological functions such as alteration of metabolic rate, glycaemia, appetite regulation, corticosterone levels and cognitive function [22–25]. To better understand how sleep deprivation affects the brain activity, some invasive procedures as the implementation of electroencephalography electrodes [26, 27], catheter insertions for drug injections and data recordings [28–30] and cerebrospinal fluid collection [31, 32] have been carried out in animal models with anesthesia. Since the use of anesthetics can promote similar alterations to those found in sleep deprivation, a prior investigation about the effects of anesthetics in the same experimental setting of the studies is desirable to avoid artifacts and misinterpretation of data.

In this study, we investigated whether KX can alter the corticosterone levels and phosphorylation degree of signaling proteins, comparing with the effects of sleep deprivation.

## Main text

### Material and methods

#### Animals

Three-month-old male Wistar rats were obtained from Center for Development of Experimental Models for Medicine and Biology (CEDEME). Animals were housed in a temperature-controlled room at 22 ± 2 °C with a 12:12 h light/dark cycle with lights on at 07 h 00 a.m. Each cage containing four animals was arbitrarily assigned to one of the three experimental: control animals that received intraperitoneal 0.9% saline before euthanasia (CT, n = 4); ketamine/xylazine animals that received intraperitoneal ketamine (90 mg/kg) and xylazine (10 mg/kg) before euthanasia (KX, n = 4) and sleep deprived animals that were subjected to paradoxical sleep deprivation for 96 h (SD, n = 4). In this method animals are placed in a water tank containing circular platforms with shallow water. Animals can move freely between the platforms but can not sleep on them, because when animals enter in paradoxical sleep, they fall into the water due to muscle atonia and are awaken [33]. Approximately 20 min before euthanasia, animals from KX group were anesthetized. The effect of anesthesia was evaluated through the animal’s response to pressure exerted on the dorsal surface of its paw. Water and food were supplied ad libitum throughout the experiment. Food was removed 2 h before euthanasia. All animals were euthanized by decapitation. Blood was collected rapidly after decapitation. After blood clotting, samples were centrifuged at 1100×*g* for 10 min and serum was collected. Hippocampi were rapidly dissected, frozen on dry ice and stored at − 80 °C. Animals from CT and SD groups were euthanized between 09 h 00 and 12 h 00, while animals from KX group were euthanized at 13 h 00. No exclusion criteria were predetermined, and none of the animals died or were excluded during the experimental period.

#### Western Blot

Hippocampi were homogenized in lysis buffer. The lysate was centrifuged for 5 min at 2700×*g* and 4 °C and the supernatant was collected. Proteins were fractionated using SDS-PAGE and transferred to PVDF membranes that were incubated with 5% of bovine serum albumin (BSA) and subsequently with specific primary antibody for 1 h at room temperature or overnight at 4 °C. After three washes with TBS-T, membranes were incubated with peroxidase-conjugated secondary antibody for 1 h, and then washed five times. Signals were developed using Luminata Forte Western HRP substrate. Images were acquired using UVITEC Imaging System (Cambridge; Alliance mini 4 m). For analysis, a square was drawn around the band of interest and the signal intensity was obtained using UVIband Image Quantification Software. The band intensity of the phosphorylated proteins was normalized by respective total protein intensity. Detailed reagents used, example given of data analysis and the uncropped western blot images can be found in Additional file 1: Table S1, Additional file 2: Data analysis, Additional file 3: Figs. S1–S4.

#### Enzyme linked immunosorbent assay (ELISA)

Corticosterone levels were measured on serum of animals following the manufacturer’s instructions using the ELISA kit. This assay is based on the binding competition for an antibody that recognizes corticosterone. Samples were incubated with a solution containing a polyclonal antibody that recognizes corticosterone to bind with a secondary antibody previously adsorbed on the plate. Then, the samples and corticosterone conjugated with horseradish peroxidase (HRP) were added to the wells to compete for binding to a specific binding site on the polyclonal antibody. After binding, substrate solution was added to determine the enzymatic activity of the peroxidase. Stop solution was then added for an absorbance reading at 450 nm.

#### Statistical analysis

For the graphical analysis of corticosterone levels and protein expression by western blot, individual data were plotted with respective group mean difference compared with CT group. Bootstrap 95% confidence interval (95CI) for group mean difference was calculated using the DABEST package implemented in a web application framework [34]. Authors were not blinded to perform the experiments and to analyze the data. Sample size was empirically determined.

### Results

**Fig. 1:**
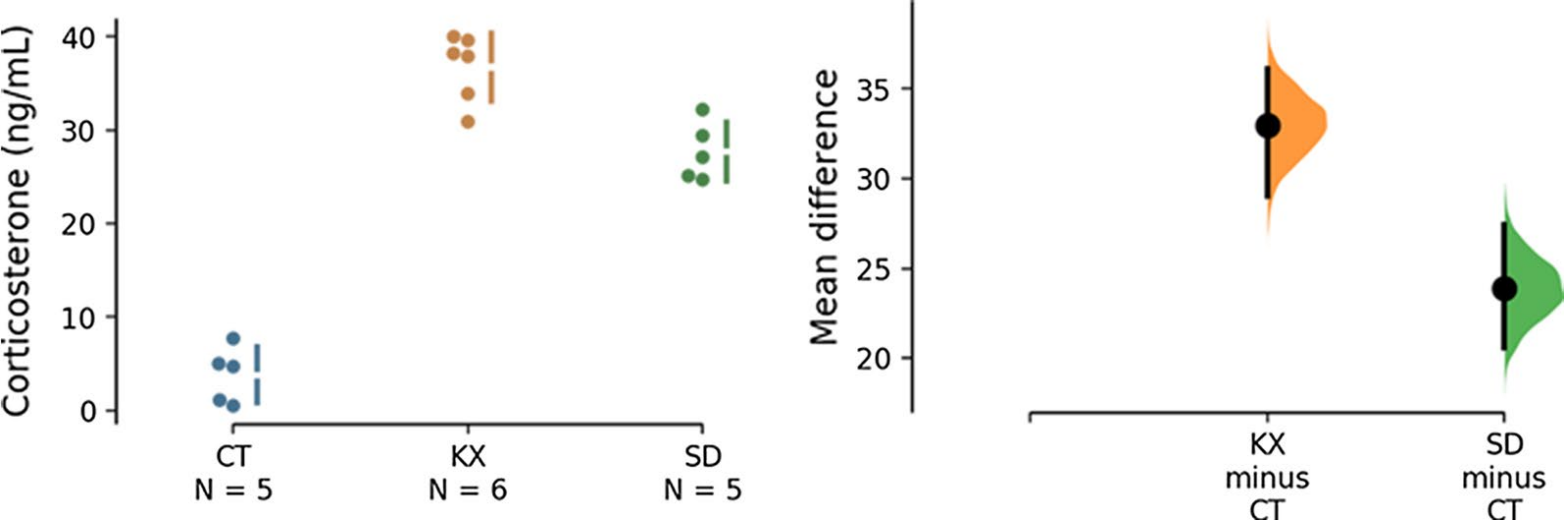
Corticosterone levels. **A** Corticosterone levels (ng/mL) measured in serum from the three experimental groups: the control group that received intraperitoneal 0.9% saline before euthanasia (CT), the anesthesia group that received intraperitoneal xylazine/ketamine before euthanasia (KX), and the sleep deprived animals (SD). Each dot plotted on the graph represents an individual rat. **B** The mean difference between the designated groups in corticosterone levels was plotted as a bootstrap sampling distribution and is depicted as a black dot. Vertical error bar represents 95CI. The unpaired mean difference between the CT and KX groups was 33.0 [95CI 29.2, 36.2] and between the CT and SD groups was 23.9 [95CI 20.7, 27.4]

In animals that received intraperitoneal injection of ketamine/xylazine (KX) prior to euthanasia higher corticosterone levels were observed when compared to animals that received saline (CT) (Fig. 1). This increase was comparable to the increase caused by sleep deprivation (SD).

Increased corticosterone levels can affect the expression of proteins related to cell death, and the excess of this hormone can induce neuronal damage [35, 36]. We, therefore, further analyzed how KX could affect some signaling molecules involved in cell survival and commonly studied in neuroscience such as p44/42 MAP kinase (ERK1/2), Ca^2+^/calmodulin-dependent protein kinase II (CAMKII) and Src family kinase.

**Fig. 2:**
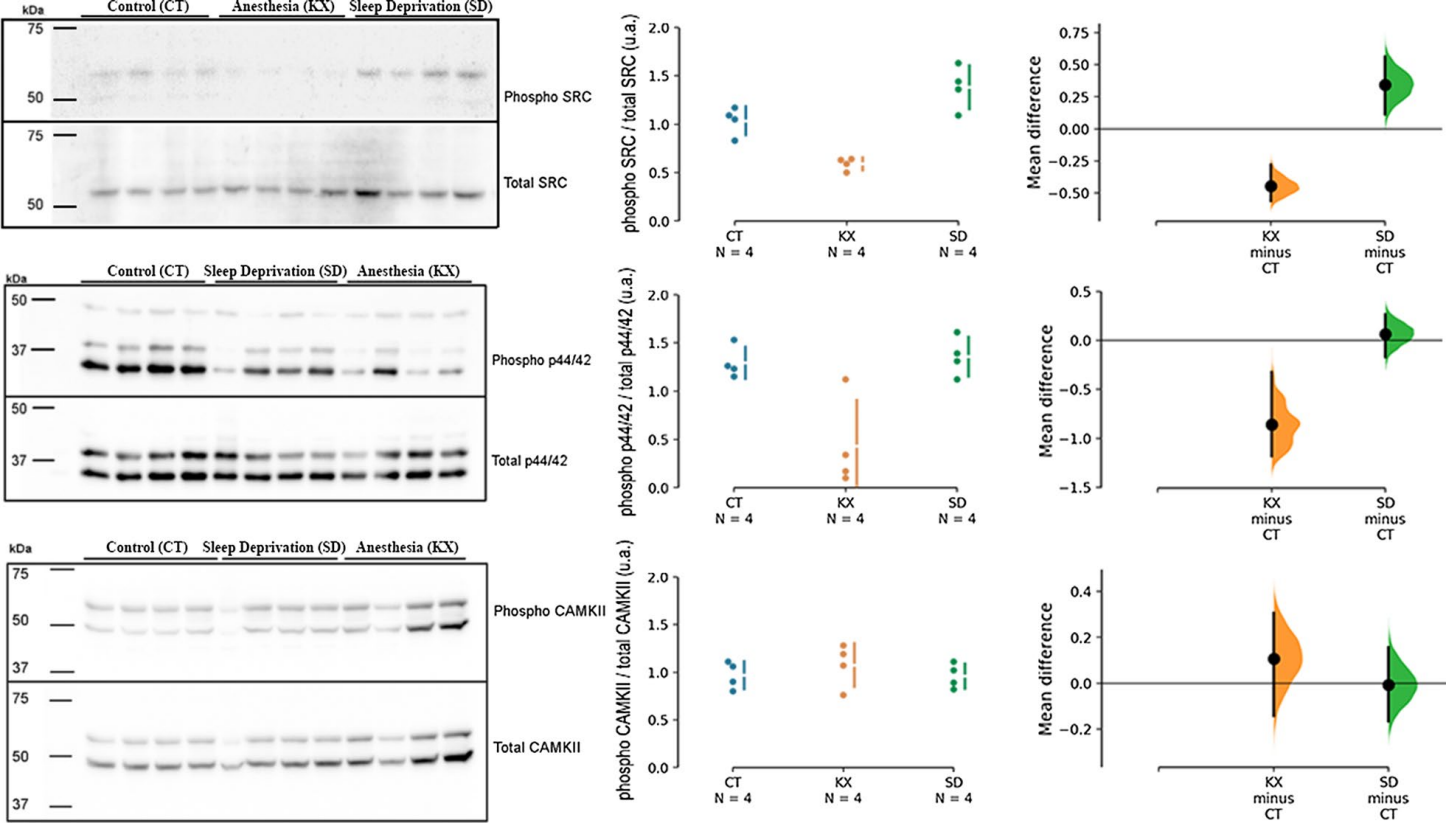
Signaling molecules expression. **A**, **D**, **G** Representative image of western blots for phosphorylated and total SRC family kinases, p44/42 MAPK and CAMKII. **B**, **E**, **H** Relative ratio of phosphorylated proteins to total proteins. Each dot represents an individual rat (n = 4 for CT, KX and SD groups). **C** The mean difference between designated groups in the degree of phosphorylation of SRC was plotted as a bootstrap sampling distribution and is depicted as a black dot. Vertical error bar represents 95CI. The unpaired mean difference between the CT and KX groups was − 0.445 [95CI − 0.56, − 0.282] and between the CT and SD groups was 0.345 [95CI 0.115, 0.562]. **F** The mean difference between designated groups in the degree of phosphorylation of p44/42 MAPK was plotted as a bootstrap sampling distribution and is depicted as a black dot. Vertical error bar represents 95CI. The unpaired mean difference between the CT and KX groups was − 0.86 [95CI − 1.18, − 0.325] and between the CT and SD groups was 0.065 [95CI − 0.173, 0.263]. **G** The mean difference between designated groups in the degree of phosphorylation of CAMKII was plotted as a bootstrap sampling distribution and is depicted as a black dot. Vertical error bar represents 95CI. The unpaired mean difference between the CT and KX groups was 0.108 [95CI − 0.143, 0.305] and between the CT and SD groups was − 0.0075 [95CI − 0.165, 0.155]

The Src family kinases acts on cell growth and differentiation [18] and p44/42 MAPK can promote cell proliferation, differentiation, mobility and cell survival [37]. The degree of phosphorylation of these proteins was diminished in animals that received anesthesia compared to the CT group. Sleep deprived animals showed similar levels of MAPK phosphorylation and slightly higher level of SRC phosphorylation than CT group (Fig. 2). These data indicate that phosphorylation of these molecules does not correlate with the corticosterone levels, since both KX and SD group showed increased corticosterone levels. CaMKII was not different between the groups (Fig. 2).

**Fig. 3:**
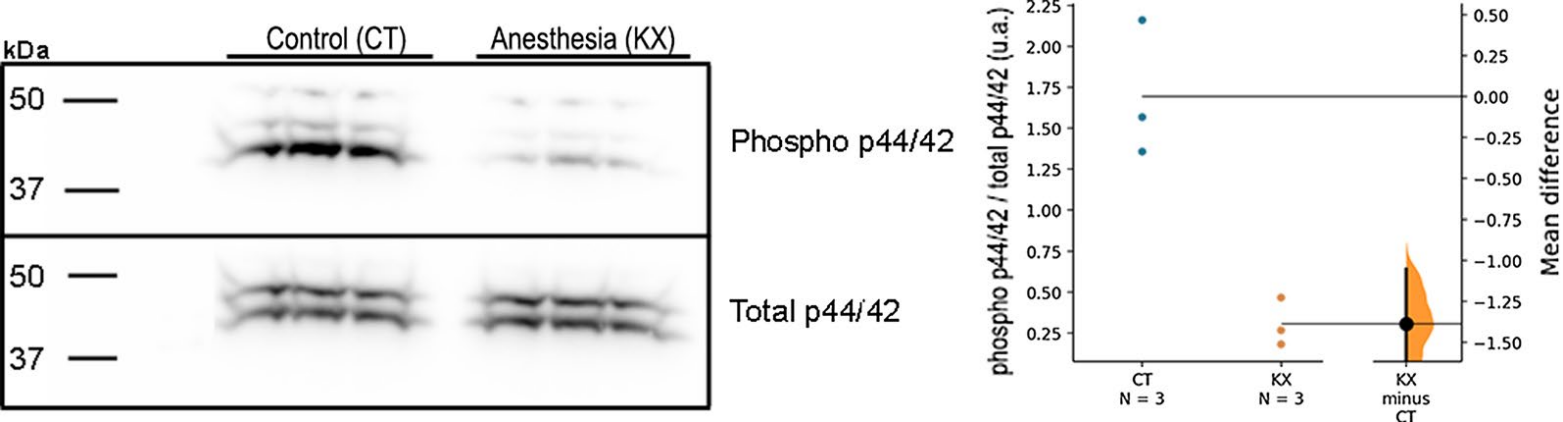
p44/42 MAP kinase expression in similar batch of animals. **A** Representative image of western blot for phosphorylated and total p44/42 MAP kinase. Each dot plotted on graph represents an individual rat. **B** Relative ratio of phosphorylated p44/42 MAP kinase to total p44/42 MAP kinase. Each dot represents an individual rat (n = 3). The mean difference between designated groups in the degree of phosphorylation of p44/42 MAP kinase was plotted as a bootstrap sampling distribution and is depicted as a black dot. Vertical error bar represents 95CI. The unpaired mean difference between the CT and KX groups was − 1.39 [95CI − 1.92, − 1.06]

One of the limitations of our study is that the samples were obtained from animals of different batches. Thus, we further verified the effect of KX on the degree phosphorylation of p44/42 MAPK in samples obtained from the same batch of animals that were euthanized in the same period of the same day, and the same results was observed with lower degree of phosphorylation in KX group (Fig. 3A, B).

### Discussion

In this study we evaluated corticosterone levels in three different groups: control animals (CT); anesthetized animals (KX) and sleep deprived animals (SD). Elevated corticosterone levels were found in both the SD and KX groups. Hohlbaum et al., claimed that the increase of corticosterone in anesthetized animals occurred due to the stress caused by the containment of the animals, and that corticosterone returned toward to basal levels after repeated applications, which may cause habituation to the procedure [38]. However, xylazine can modulate the secretion of glucoregulatory hormones in a dose-dependent manner [13, 39]. Therefore, it is reasonable to suggest that the pharmacological action of these anesthetics is intimately related to corticosterone levels, and that the stress caused by the injection may not be the only factor responsible for the increase of corticosterone.

Corticosteroids can rapidly change neuronal activity and excitability in distinct brain areas [40, 41]. We then investigated the phosphorylation of signaling molecules frequently studied in neuroscience and showed that ratios of phosphorylated to total p44/42 MAPK (ERK1/2) and SRC family kinases were reduced KX animals, while the ratio of phosphorylated to total CaMKII did not change. A recent study also showed that ketamine/xylazine can reduce the activation of ERK1/2 in mouse hippocampus [42]. However, this reduction appears to be caused in response to the thermoregulatory effect of ketamine [43, 44]. These results reinforce the need for a study design taking these effects into account.

It is known that SRC kinases acts anchored at NMDA receptor (NMDAR) complex through NADH dehydrogenase subunit 2 (ND2) [45]. Moreover, depending on the ligands that bind to SH2 domain, SRC kinases can upregulate or depresses NMDAR activity [46, 47]. Thus, SRC kinases participate in the regulation of synaptic transmission through the modulation of NMDAR activity [48, 49]. Ketamine antagonizes NMDAR and reduces the time of channel overture [49]. It is plausible that blocking NMDAR may lead to a reduction in the activation of SRC kinases by feedback mechanism, but this phenomenon needs to be further investigated.

Nonetheless, the use of SRC kinases and NMDAR modulators, as Ketamine, should be carefully evaluated, especially for studies that use animal models for epilepsy, ischemia and neurodegeneration. Animal models for epileptiform activity shows elevated hippocampal SRC activity, while its blockade reduces the frequency of epileptiform discharges [50]. In models of cerebral ischemia, phosphorylation of NMDAR is increased. The activities of NMDARs are also involved in neuronal loss [51]. Lastly, dysregulated SRC kinases and upregulation of NMDAR have been also associated with chronic pain and schizophrenia [52].

In contrast, CAMKII levels were similar between groups. CAMKII is a critical protein for the induction of long-term potentiation, triggered by Ca^2+^ entry through NMDAR [53]. Higher levels of intracellular Ca^2+^ affects several downstream signaling pathways that involve CAMKII, including its translocation to the postsynaptic density, where it binds directly to NMDAR [54]. In our study we used an antagonist for NMDAR, thus it is possible that lack of NMDAR activation resulted in maintenance of CAMKII levels.

Lastly, it is also important to consider the duration of anesthesia and long-lasting damages. Saha et al. showed that the effects of KX could last up to 180 min when a maintenance dose is applied [13]. Another study reported that rats can remain up to 300 min without righting reflex after KX anesthesia [55]. Some alterations caused by KX can be irreversible. For instance, a permanent corneal damage and the generation of keratopathy in mice [56], a muscle skeletal necrosis [57] and middle cerebral artery occlusion associated with cerebrovascular changes [58] have been reported. KX also can show long-term negative effects in synaptic plasticity, spine turnover and memory consolidation [59]. Thus, evaluation of the effects of anesthesia on the biological pathways of interest can avoid undesirable results and help to generate more reliable knowledge.

### Conclusions

This study demonstrated that peritoneal administration of ketamine/xylazine in rats alters corticosterone levels and may potentially change cellular signaling as ERK1/2 and SRC kinases, emphasizing the importance of including an evaluation of the effects of anesthetics in each study design, and taking any effect into account when interpreting the results.

## Limitations

The most significant limitation of the current research is its small sample size. Also, animals of CT and SD groups were from different batch than the KX group. The CT and SD samples originated from a study with the protocol number #0764/10, while the KX samples originated from a study with the protocol number N9806251113. Is reasonable to mention that we did not evaluate the animal temperature pre and post anesthesia, a factor that can modulate ERK1/2 levels as presented in discussion section.

## Supplementary Information

**Additional file 1: Table S1.** List of materials used in experiments.

**Additional file 2.** Data analysis on Western Blot section.

**Additional file 3: Figure S1.** Phospho and Total SRC Western Blot. Top panel shows uncropped immunodetection of phospho SRC and lower panel shows uncropped immunodetection of total SRC. Both images corresponds to cropped bands shown in Fig. 2 of the main article. **Figure S2.** Phospho and Total SRC Western Blot. Top panel shows uncropped immunodetection of phospho p44/42 MAPK and lower panel shows uncropped immunodetection of total p44/42 MAPK. Both images corresponds to cropped bands shown in Fig. 2 of the main article. **Figure S3.** Phospho and Total CAMKII Western Blot. Top panel shows uncropped immunodetection of phospho CAMKII and lower panel shows uncropped immunodetection of total CAMKII. Both images corresponds to cropped bands shown in Fig. 2 of the main article. **Figure S4.** Phospho and Total p44/42 MAPK Western Blot. Top panel shows uncropped immunodetection of phospho p44/42 MAPK and lower panel shows uncropped immunodetection of total p44/42 MAPK. Both images corresponds to cropped bands shown in Fig. 3 of the main article.

## Data Availability

The data sets used and/or analyzed during the current study are available from the corresponding author on reasonable request.

## Abbreviations

CaMKII: Ca2+/calmodulin-dependent protein kinase II
CT: Control
KX: Ketamine and xylazine
NMDAR: NMDA receptor
SD: Sleep deprivation/deprived
