# Supplementary figures and images for: Anesthesia can alter the levels of corticosterone and the phosphorylation of signaling molecules
Source: BMC Res Notes. 2021 Sep 19;14:363. doi: 10.1186/s13104-021-05763-w (PMC8451088; doi:10.1186/s13104-021-05763-w)

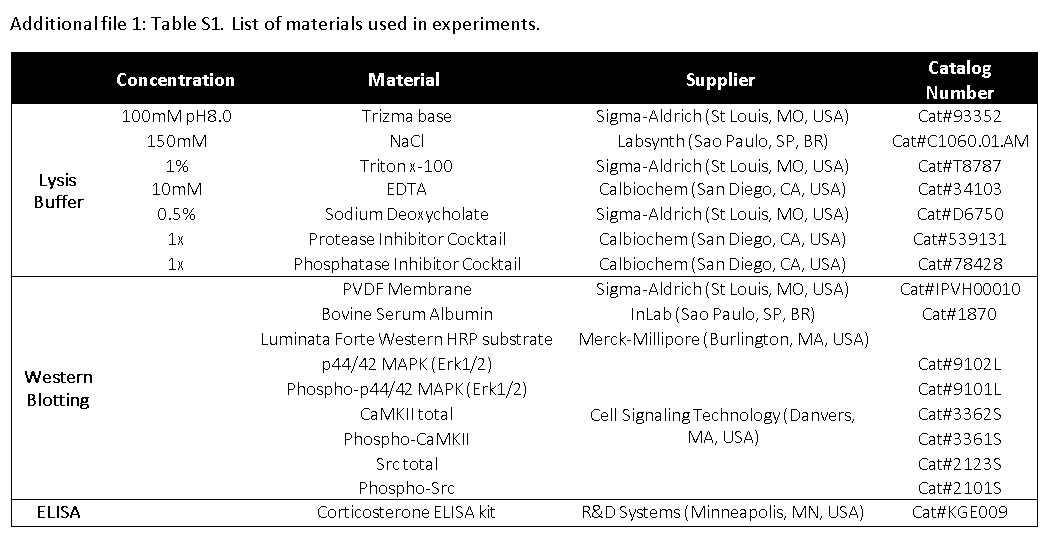

Supplement: Supplementary file 1 — Additional file 1: Table S1. List of materials used in experiments. [file 13104_2021_5763_MOESM1_ESM.tif]
