# Supplementary material for: Anesthesia can alter the levels of corticosterone and the phosphorylation of signaling molecules
Source: BMC Res Notes. 2021 Sep 19;14:363. doi: 10.1186/s13104-021-05763-w (PMC8451088; doi:10.1186/s13104-021-05763-w)
Supplement: Supplementary file 3 — Additional file 3: Figure S1. Phospho and Total SRC Western Blot. Top panel shows uncropped immunodetection of phospho SRC and lower panel shows uncropped immunodetection of total SRC. Both images corresponds to cropped bands shown in Fig. 2 of the main article. Figure S2. Phospho and Total SRC Western Blot. Top panel shows uncropped immunodetection of phospho p44/42 MAPK and lower panel shows uncropped immunodetection of total p44/42 MAPK. Both images corresponds to cropped bands shown in Fig. 2 of the main article. Figure S3. Phospho and Total CAMKII Western Blot. Top panel shows uncropped immunodetection of phospho CAMKII and lower panel shows uncropped immunodetection of total CAMKII. Both images corresponds to cropped bands shown in Fig. 2 of the main article. Figure S4. Phospho and Total p44/42 MAPK Western Blot. Top panel shows uncropped immunodetection of phospho p44/42 MAPK and lower panel shows uncropped immunodetection of total p44/42 MAPK. Both images corresponds to cropped bands shown in Fig. 3 of the main article. [file 13104_2021_5763_MOESM3_ESM.pdf]

Additional file 3: Figs. S1–S4. Original Western Blot images.

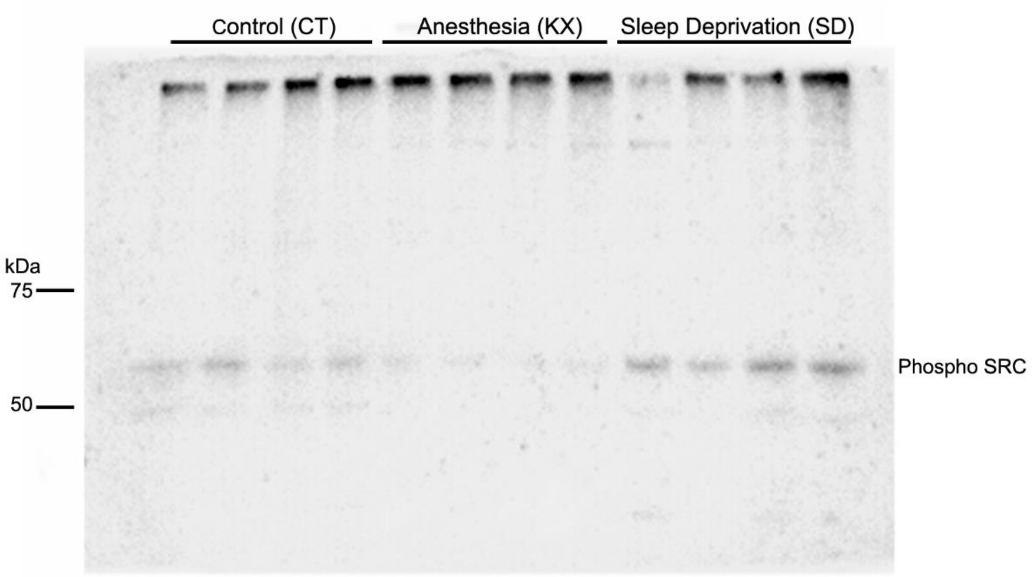

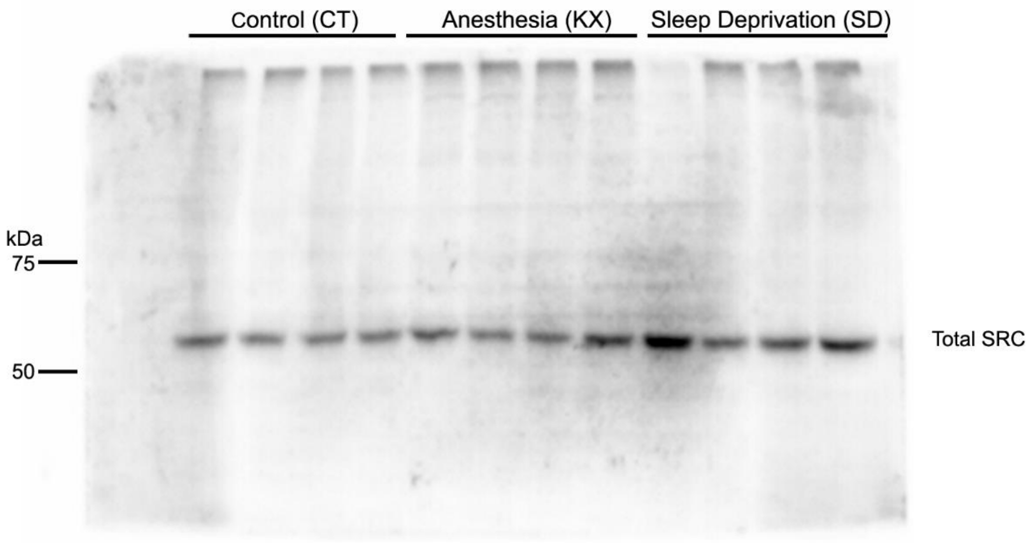

Supplementary Figure 1. Phospho and Total SRC Western Blot. Top panel shows uncropped immunodetection of phospho SRC and lower panel shows uncropped immunodetection of total SRC. Both images corresponds to cropped bands shown in figure 2 of the main article.

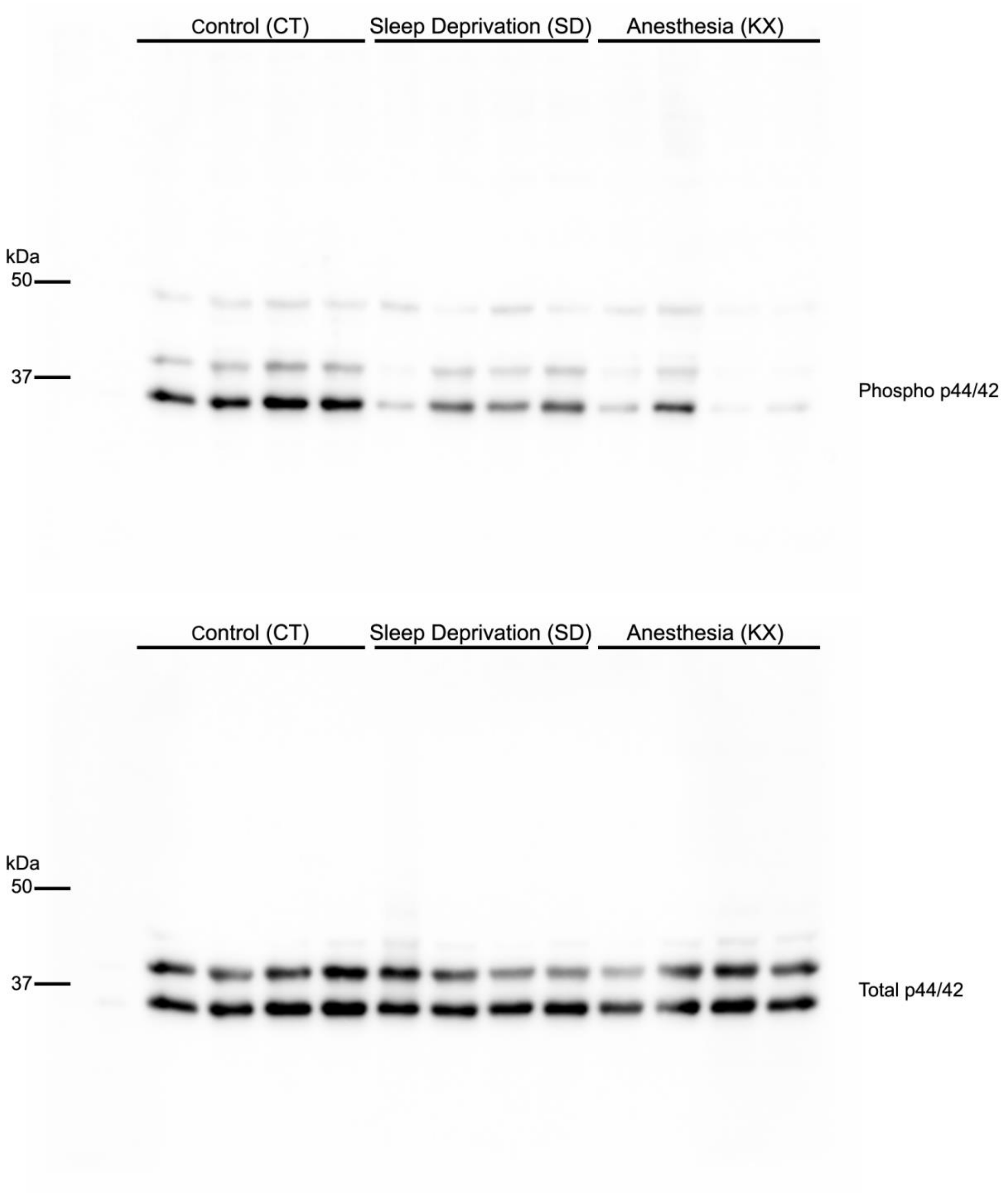

Supplementary Figure 2. Phospho and Total SRC Western Blot. Top panel shows uncropped immunodetection of phospho p44/42 MAPK and lower panel shows uncropped immunodetection of total p44/42 MAPK. Both images corresponds to cropped bands shown in figure 2 of the main article.

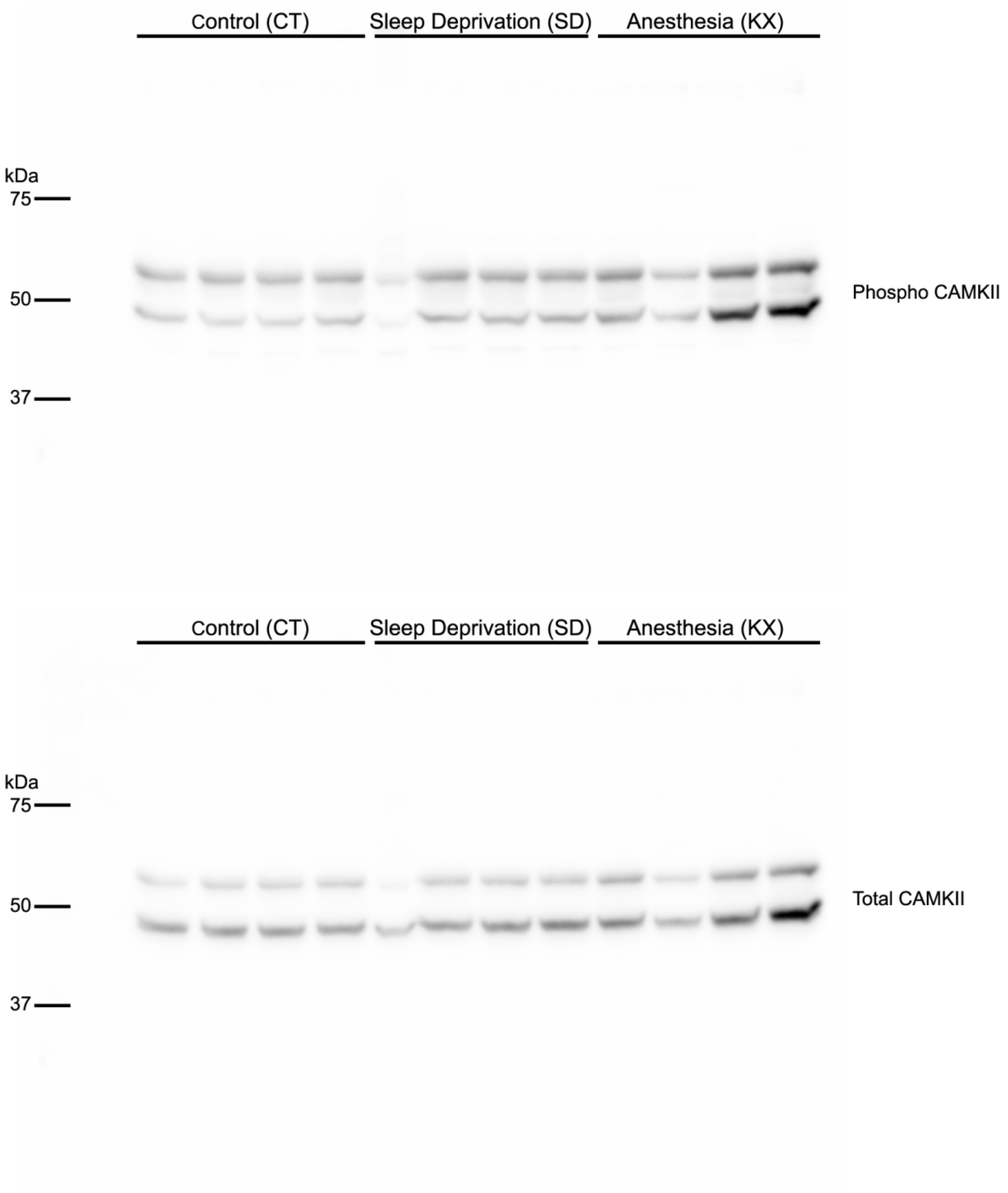

Supplementary Figure 3. Phospho and Total CAMKII Western Blot. Top panel shows uncropped immunodetection of phospho CAMKII and lower panel shows uncropped immunodetection of total CAMKII. Both images corresponds to cropped bands shown in figure 2 of the main article.

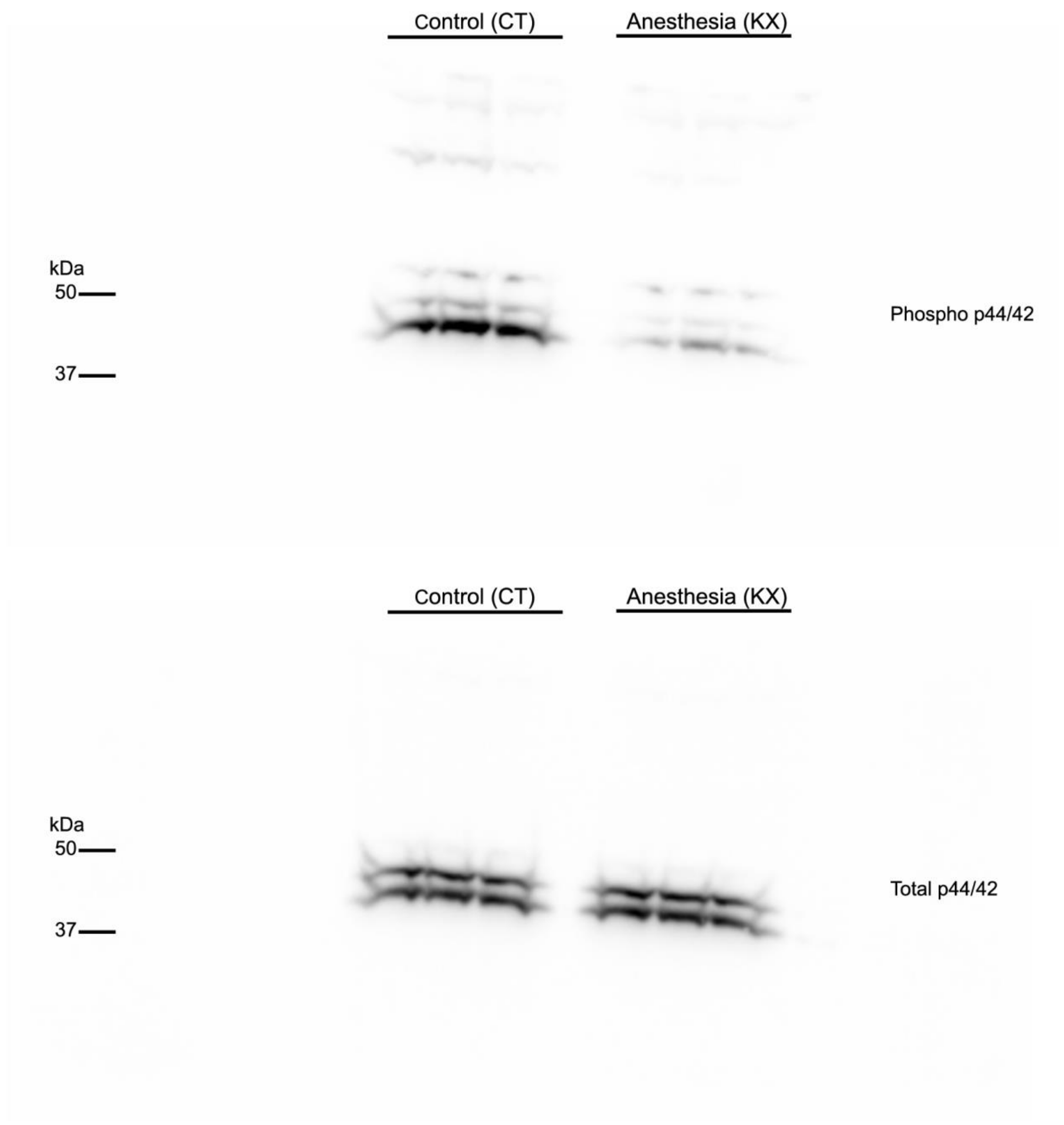

Supplementary Figure 4. Phospho and Total p44/42 MAPK Western Blot. Top panel shows uncropped immunodetection of phospho p44/42 MAPK and lower panel shows uncropped immunodetection of total p44/42 MAPK. Both images corresponds to cropped bands shown in figure 3 of the main article.
